# Longitudinal development of language and fine motor skills is correlated, but not coupled, in a childhood atypical cohort

**DOI:** 10.1177/13623613221086448

**Published:** 2022-04-26

**Authors:** Marie K Deserno, Delia Fuhrmann, Sander Begeer, Denny Borsboom, Hilde M Geurts, Rogier A Kievit

**Affiliations:** 1 Dr. Leo Kannerhuis and REACH-AUT, The Netherlands; 2 University of Amsterdam, The Netherlands; 3 Max Planck Institute for Human Development, Germany; 4 University of Cambridge, UK; 5 King’s College London, UK; 6 Vrije Universiteit, The Netherlands

**Keywords:** language development, longitudinal cognitive dynamics, mutualistic coupling, structural equation modeling

## Abstract

Autism is often associated with early developmental delays in language and motor skills. However, little is known about the complex dynamic processes that drive the co-development of such early difficulties. The aim of the present study was to model the parallel growth of language and motor skills in a cohort of infants and to explore differences between infants with typical development and those with atypical development. Receptive and expressive language and fine motor skills were repeatedly assessed in a group of 239 infants (7 months at t1 and 36 months at t4) from the British Autism Study of Infant Siblings sample. Latent Growth Curve Analysis was applied to investigate the mutualistic coupling of longitudinal changes in these domains. Our results showed highly correlated slopes but we did not find an association between baseline scores in one domain and rates of change in the other (i.e. coupling). In the later diagnosed group, we found that scores at baseline and rates of change were more variable.

**Lay abstract:**

More and more members of the autistic community and the research field are moving away from the idea that there will be a single biological or cognitive explanation for autistic characteristics. However, little is known about the complex dynamic processes that could explain why early difficulties in the language and motor domain often go hand-in-hand. We here study how language and motor skills develop simultaneously in the British Autism Study of Infant Siblings cohort of infants, and compare the way they are linked between children with and without developmental delays. Our results suggest that improvements in one domain go hand-in-hand with improvements in the other in both groups and show no compelling evidence for group differences in how motor skills relate to language and vice versa. We did observe a larger diversity in motor and language skills at 6 months, and because we found the motor and language development to be tightly linked, this suggests that even very small early impairments can result in larger developmental delays in later childhood. Greater variability at baseline, combined with very strong correlations between the slopes, suggests that dynamic processes may amplify small differences between individuals at 6months to result into large individual differences in autism symptomatology at 36 months.

Classical psychometric approaches implicitly consider cognitive development as a linear system. However, emerging work suggests that a complex systems approach might be better suited for capturing developmental phenomena such as equilibration (e.g. refining mental structures; Piaget, 1971), self-organization (in the domain of learning; Kohler, 1940; Lorber et al., 2014), or emergence (of higher-order cognitive skills; Anderson, 2008). The complex systems toolbox aims to capture the interaction between genetic, physiological, and social factors that contribute to typical or atypical brain and behavioral development. In the current study, we investigate behavioral cross-domain coupling in infants with and without elevated likelihood (EL) for atypical development.

“Coupling” is a term commonly used (e.g. McArdle, 2009, p. 597) in longitudinal structural equation models (SEMs) and captures the extent to which growth in one domain is governed by the starting point in another. A promising avenue to pursue from this perspective is the idea that observed heterogeneity in autism is a result of small differences amplifying to produce large differences in emergent phenotypes (Johnson, 2017), similar to the heterogeneity we observe in typically developing individuals. This interpretation is analogous to the mutualism model (Van der Maas et al., 2006), which proposes a network of multiple interacting and mutually reinforcing factors contributing to development of cognitive abilities. Notably, simulations demonstrate how small dynamic effects in these mutualistic causal pathways can amplify over time, and lead to developmental discontinuities. Note that “coupling” refers to a general single parameter in a particular type of SEM (the latent change score model, for example, McArdle, 2009, p. 597), whereas “mutualism” captures a modeling framework which is based on the positive manifold (i.e. most parameters are positive) drawn originally from ecology, but in psychology commonly used to study general cognitive ability/intelligence (Kievit et al., 2017; Van der Maas et al., 2006).

One of the first empirical investigations of mutualism has looked at the co-development of fluid reasoning and vocabulary (Kievit et al., 2017). The authors found strong support for the idea that variation in these cognitive domains arises through their mutual coupling. Individuals with higher initial scores in vocabulary show greater gains on matrix reasoning over time and vice versa (Kievit et al., 2017, with Kievit et al., 2019, showing a replication with even stronger effects in younger children). A similar approach to the developmental interrelation between vocabulary knowledge and reading comprehension has shown one-way coupling, that is, vocabulary knowledge acts as a driving force for an individual’s gains in reading comprehension, but not vice versa (Quinn et al., 2015).

These examples show that the mutualism model is a useful empirical framework for investigations of the longitudinal dynamics in typical development. In addition, simulation work (e.g. Van der Maas et al., 2006) demonstrates that profound differences in phenotypes may arise purely from disruptions to the dynamic system, rather than deficits within a narrow domain. For example, Baughman and Thomas (2008) simulated different types of disruptions to the mutualism model, and their results suggested that the effect of early impairments depends on the connectivity (the number and strength of mutually connected domains) and the time-sensitive centrality (how relevant is the disrupted domain to other developing domains) of the targeted domain. This highlights the importance of studying how developmental delays can “spread” through a highly connected system of developing skills. Given the magnitude of these effects, it may be plausible that other conditions characterized by atypical development, such as autism, may also be characterized by different dynamic interactions between developmental domains. Some evidence exists that coupling may be disrupted in specific populations. One study suggested that the developmental *un*-coupling of cognition and reading might be the source of learning disability in the case of readers with dyslexia (Ferrer et al., 2010). Typical readers showed bidirectional coupling, that is, higher IQ, predicted greater gains in reading comprehension, and vice versa. In dyslexic readers, mutualistic coupling was much smaller (non-significant), suggesting that the general cognitive skills of readers with dyslexia did not increase as quickly with greater reading, nor did their reading ability benefit from general cognitive developments to the same extent as typically developing individuals. To the best of our knowledge however, such approaches have not yet been used to study autistic development.

Autism is clinically defined based on impaired social-communication skills together with restricted and repetitive interests (American Psychiatric Association (APA), 2013) and is often associated with developmental delays in both language and motor domains. The coupled development of these domains has been shown in typically developing children (Iverson, 2010; Leonard & Hill, 2014), and empirical evidence highlights the predictive association between impairments in infant motor functioning and autism-related impairments at a later developmental stage (Bedford et al., 2016; Brian et al., 2008; Choi et al., 2018; Leonard et al., 2014, 2015). This link has been proposed to result from increasing social learning opportunities depending on the range of motor skills, such that infants able to gesture or C-walk were more likely to get a response from their mother compared to crawling infants (Choi, Leech, Tager-Flusberg & Nelson, 2018; Karasik et al., 2014). This suggests that fine motor skills influence the frequency of the infant’s interactions with people around them, which in turn facilitates their language learning. Prior research on such mutual reinforcement between fine motor and language, however, remains scarce. Although some previous studies have explored longitudinal trajectories of these domains (e.g. Choi et al., 2018; Leonard et al., 2015), such studies are still relatively rare.

In the current study, our aim was to investigate how the co-development of language and motor skills can inform us about dynamic processes that drive atypical development. In the long run, such informed longitudinal models could enable us to detect developmental challenges at an early stage and intervene, when deemed appropriate, before they self-reinforce over time. We here concentrate on the fine motor domain, given that the link for gross motor has been established in a previous study (Leonard et al., 2015) but fine motor is necessary for gesturing and therefore relevant for social interaction and language (Mody et al., 2017). One previous paper examined a subset (N = 101) of the present sample to study the association between baseline gross motor skills and longitudinal language trajectories (see Leonard et al., 2015), and found that higher baseline gross motor abilities were correlated with more rapid gains in language ability. The Leonard et al. (2015) study specifically examined growth in language as predicted by early gross motor skills (for more detail, see below). We move beyond this work by (a) analyzing a considerably larger sample (N = 239 vs N = 101), (b) modeling *growth* in both domains (motor and language), (c) estimating group differences in all pathways (including variances), (d) modeling direct interactions between the domains as well as correlated slopes using a *multigroup parallel process model*, and (e) focusing on fine motor skills. We hypothesize that there is significant cross-domain coupling between starting points and growth rate of language and motor skills. We also applied multigroup growth curve model to investigate whether those infants who develop atypically differ in their rate of change and co-development of these skills from those who do not.

## Methods

### Sample descriptives

Participants were infants taking part in the British Autism Study of Infant Siblings (BASIS, www.basisnetwork.org), an ongoing longitudinal research program aimed at monitoring early development of infants with siblings diagnosed with autism compared to infants without an autistic sibling. For further details of recruitment and sample characteristics, see Elsabbagh et al. (2013) for the first recruitment wave of BASIS and Green et al. (2015) for the second recruitment wave. Ethical approval for this specific study was obtained from the World Health Organization (WHO), 1993. As part of the BASIS study, 250 infants completed a battery of assessments at 6–9 months (n_t=1_ = 238), 14 months (n_t=2_ = 233), 24 months (n_t=3_ = 221), and 36 months of age (n_t=4_ = 228). We included all 239 participants (118 boys, 121 girls) who completed at least three assessments of the Mullen Scales of Early Learning (MSEL; Mullen, 1995) and the Autism Diagnostic Observation Schedule–Generic (ADOS-G; Lord et al., 2000), and whose diagnostic outcome at 36 months was known.

### Group comparison

The study design included two groups of children: one group with typical likelihood (TL) for atypical development, that is, with a typically developing older sibling, and one group with EL of autism, that is, who had an older sibling with an Autism Spectrum Disorder (ASD) diagnosis. At a later phase, the children were split into two groups (distinct from the a priori TL-EL division) based on their clinical outcome at 36 months—those who met ASD criteria or had some subclinical symptoms or low IQ (defined as “atypical”) and those with typical development (note: children, regardless of their a priori TL or EL label, who did not end up receiving a clinical diagnosis were thus considered to be “typically developing” in our grouping). In order to determine who was part of the atypical or typical group, expert clinical researchers reviewed all information gathered about infants at the 24- and 36-month assessments (including MSEL, ADOS, and Vineland Adaptive Behavior Scales (VABS; Sparrow et al., 1984); see Gammer et al., 2015, for more details). These experts then decided on the best estimate diagnosis according to the *Diagnostic and Statistical Manual of Mental Disorders* (5th ed.; DSM-5) criteria (APA, 2013) and International Classification of Diseases (ICD-10) criteria (World Health Organization (WHO), 1993). Based on these diagnostic classifications, we here split the sample into two subgroups based on their clinical outcome at 36 months: 74 atypically developing infants and 165 typically developing infants (note that this includes EL infants who did not develop symptoms). Since we were interested in potential differences in longitudinal dynamics of language and motor development between those who eventually develop an atypical developmental profile versus those who do not (regardless of their at-risk label of EL or TL), this is the grouping we used for the comparative analyses in the current study. If one were to assume categorical differences between those with autism-specific developmental delays and those with other developmental impairments, the autism diagnosis would present a better suited grouping variable. Of the atypical group, 34 infants were diagnosed with autism and 39 with other developmental delays and symptoms. See Table 1 of the supplement for both demographics and questionnaire scores reported for each group (atypical vs typical) at every assessment point and Bussu et al. (2019) for more details on classification and general sample descriptive statistics for the BASIS sample. Note that a small subgroup of the children received intervention (n = 34) in a randomized controlled trial (Green et al., 2015). They were balanced equally across the atypical versus typical outcome group of the current study (N_typical = 19, N_atypical = 14, N_na = 1). Data from the BASIS project can be requested through the BASIS project email address (basis@bbk.ac.uk). All scripts used are publicly available at https://osf.io/en4xj/.

### Measures

We focused on two subscales of the MSEL (Mullen, 1995), a widely used and well-validated measure of cognitive functioning for children with developmental disabilities (Bishop et al., 2011, and Lord et al., 2006; but see Akshoomoff, 2006, for some cautionary notes regarding testing in atypical populations). The MSEL are a standardized test for testing receptive and expressive language, visual reception, and gross and fine motor skills for the age range of 0–68 months. The MSEL was administered by a clinician in the presence of the infant’s parent. Since we were interested in the global growth trend in individual growth curves instead of relative rank slopes, we used raw scores of both language subscales (Receptive and Expressive) and the Fine Motor subscale. We did not include the Gross Motor subscale since it was not assessed after 24 months. The Fine Motor subscale spans 33 items assessing skills ranging from evidence of reflexes to drawing a triangle. The Receptive Language subscale has 33 items assessing skills ranging from comprehension, memory, and reflexes to noise. The Expressive Language subscale is comprised of 28 items assessing vocabulary and word semantic skills.

### Modeling framework

The trajectories of the MSEL domains were then modeled using latent growth curve models (LGMs). Models were estimated using the R-package *lavaan* version 0.6-1 (Rosseel, 2012) in R version 3.4.0 (“You Stupid Darkness”). Fitting LGMs allowed us to identify an appropriate growth curve for domain development over time. We fit a latent-basis model as this is the most flexible model if linear or polynomial change may be too restrictive (McArdle, 2009; Stoel et al., 2004): by only constraining the first and last factor loadings, it allows for capturing a wide range of non-linear shapes. In our models, the slope factor loadings were freely estimated for timepoint 2 (14 months) and timepoint 3 (24 months) for both language domains and the motor domain. This implementation, known as “latent-basis” coefficients (McArdle, 2009), is preferred here since (a) we had no a priori hypotheses about the rate of change in these domains and (b) we think it unlikely development will be purely linear. We use robust maximum likelihood estimator with a (Yuan–Bentler) scaled test-statistic and robust (Huber–White) standard errors to account for deviations from multivariate normality. In a second step, we fit a multigroup growth curve model to test for group differences in individual parameters of the model between the atypical (defined at age 36 months) group and the typically developing group. We used the full information maximum likelihood estimator to account for missingness. To assess model fit, we inspected the comparative fit index (CFI), the root mean square error of approximation (RMSEA), and the standardized root mean squared residual (SRMR). These indices are usually interpreted as follows (Schermelleh-Engel et al., 2003): CFI (acceptable 0.95–0.97, good > 0.97), RMSEA (acceptable < 0.08, good < 0.05), SRMR (acceptable 0.05–0.10, good < 0.05); however, we note that LGMs, especially with small or modest sample sizes, often display poorer absolute model fit even when the true model is estimated (e.g. DeRoche, 2009). Many valuable tutorial resources for longitudinal SEM exist, for instance, those published by Duncan and Duncan (2004), McArdle (2009), and Newsom (2015) or the online tutorials offered by the QuantDev group from Pennsylvania State University. There was no community involvement in the reported study.

## Results

**Figure 1.**
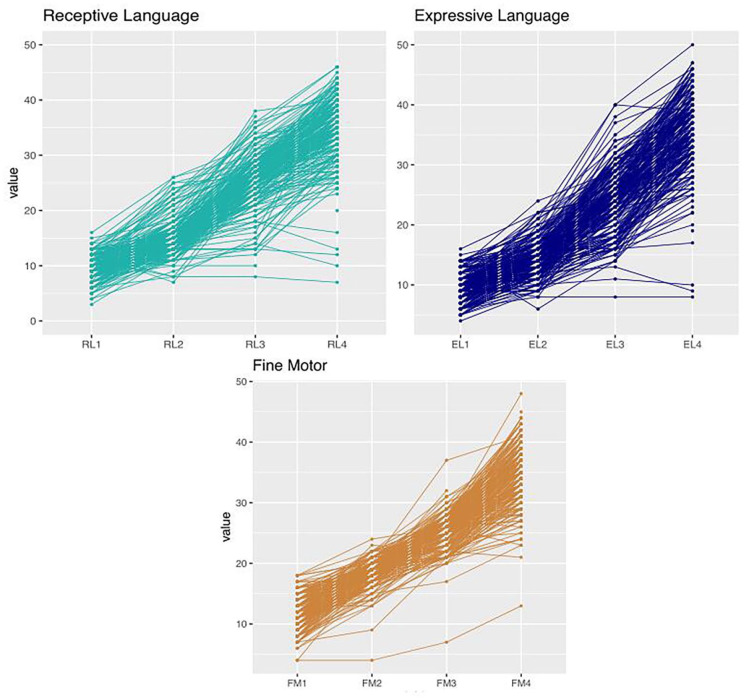
Trajectories for Fine Motor (FM), Receptive Language (RL), and Expressive Language (EL) development (Mullen Scale of Early Learning) over four assessments at (on average) 8, 14, 24, and 36 months of age in 239 children with and without elevated likelihood for atypical development.

Figure 1 shows the domain-specific trajectories for the raw data for the complete sample (N = 239) on the Fine Motor subscale of the MSEL (bottom), the Expressive Language subscale (upper right), and the Receptive Language subscale (upper left).

### Language and fine motor LGM

Since we were interested in potential dynamic relations between the co-development of language and motor skills, we modeled their growth trajectories simultaneously: First for expressive language, then for receptive language. First, in order to analyze the mean growth trajectories of these domains, we fit a *parallel process model* (Muthén & Muthén, 2005, example 6.13, or Kievit et al., 2019, Figure 2) to the full sample with four assessments of children with and without EL for atypical development. This model regresses the slope of one domain on the intercept of the other domain. Coupling would manifest as higher intercepts in one domain being associated with greater (or less, if the parameter estimate is negative) gains in the other domain. This latent growth curve approach often yields more reliable convergence than similar models such as the dual change score model (Kievit et al., 2018; McArdle, 2009) and as such is more suitable for modeling an atypical sample with a moderate sample size. Notably, this parallel process model can capture similar coupling effects as the latent change score model (Kievit et al., 2019, Figure 2).

**Figure 2.**
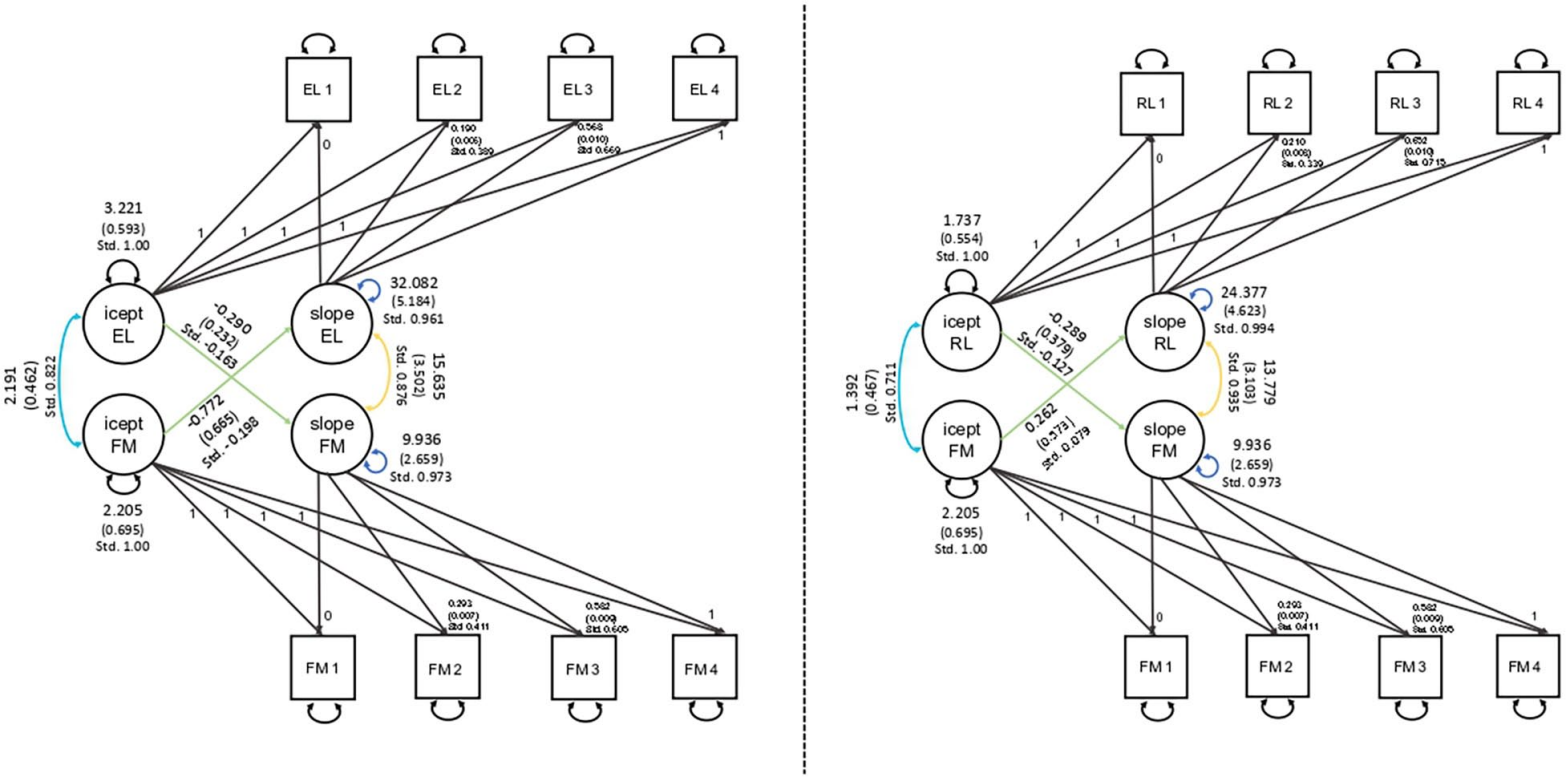
Parallel process models for Expressive Language (EL) and Fine Motor (FM), and Receptive Language (RL) and Fine Motor (FM) with freely estimated slope factor loadings at 14 and 24 months, error variances, and structured residuals. Latent variables such as the intercepts (icept) and slopes (slope) are shown as circles, and observed variables are represented by rectangles (with numbers 1–4 referring to the respective measurement occasion). Error variances and structured residuals were allowed to differ over time to allow for time-specific growth effects.

The parallel process model (see Figure 2) fit to the full sample showed acceptable fit: χ^2^(16) = 37.66, p = 0.002; CFI = 0.966; RMSEA = 0.074; SRMR = 0.068.

**Figure 3.**
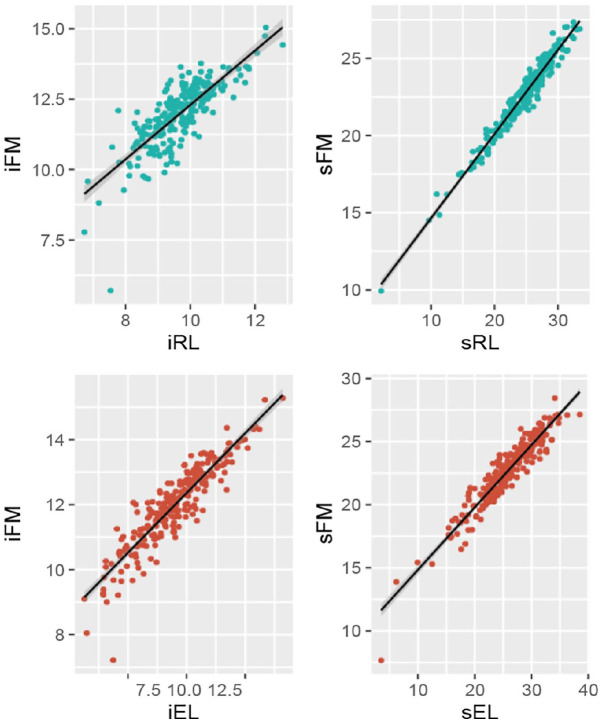
Cross-domain intercept–intercept (left column) and slope–slope correlations (right column) between Receptive Language (RL) and Fine Motor (FM) and Expressive Language (EL) and Fine Motor (FM).

There was significant variation in the intercepts of both language domains and motor skills, indicating individual differences in baseline levels of these developmental domains at timepoint 1 (corresponding to an average age of 7 months). There was significant growth in both language and motor skills as well as significant variation in the growth trajectories of these domains, as indicated by the slope estimates (Table 1). Most strikingly, the correlation between the slope parameters (Table 1) was extremely high (0.935/0.876). In other words, as can be seen by visual inspection of the slope–slope estimates in Figure 3, more rapid gains in language (especially receptive language) were almost perfectly associated with more rapid growth in the fine motor skills, suggesting almost isomorphic co-development of motor skills and both expressive and receptive language skills. However, contrary to our hypothesis, we did not find significant cross-domain coupling between intercepts and slopes of language and motor skills.^1^ This indicates that, in the current sample, there is no significant driving effect of one of these domains on the development of the other over time.

**Table 1.** (a) Group-level parameters (intercept (i) and slope (s)) for the parallel process model with Receptive Language (RL) and Fine Motor (FM). (b) Group-level parameters (intercept (i) and slope (s)) for the parallel process model with Expressive Language (EL) and Fine Motor (FM).

(a) Receptive Language and Fine Motor			
Parameters	Estimate		p
iRL	9.688		<0.0001
sRL	24.737		<0.0001
iFM	11.995		<0.0001
sFM	22.689		<0.0001
Covariances	Raw	Std.	p
sRL ~~ sFM	13.779(3.013)	0.935	<0.0001
iRL ~~ iFM	1.392(0.467)	0.711	<0.01
Regressions	Raw	Std.	p
sRL ~~ iFM	0.262(0.573)	0.079	0.648
sFM ~~ iRL	−0.289(0.379)	−0.127	0.445
(b) Expressive Language and Fine Motor			
Parameters	Estimate		p
iEL	9.497		<0.0001
sEL	35.122		<0.0001
iFM	11.988		<0.0001
sFM	25.452		<0.0001
Covariances	Raw	Std.	p
sEL ~~ sFM	15.635 (3.502)	0.876	<0.0001
iEL ~~ iFM	2.191 (0.462)	0.822	<0.0001
Regressions	Raw	Std.	p
sEL ~~iFM	−0.772 (0.665)	−0.198	0.246
sFM ~~ iEL	−0.290 (0.232)	−0.163	0.211

### Multigroup growth curve model

In a second step, we tested for differences and similarities between children that later receive a diagnosis (n = 74) and those who do not (n = 165) by testing multigroup LGMs. In these model comparisons, we tested for group differences in specific parameters while constraining all other parameters in the model to be equal across the two groups. We start out with a model in which all parameters are equality constrained. If this is an adequate approximation, model comparison will prefer such a simpler model. If the model does not seem to have adequate fit, we can subsequently free specific (groups of) parameters to examine whether estimating them independently for both groups leads to an improvement in fit greater than expected by chance in which case we can assume that the two groups differ on the relevant parameter of interest.

This succession of model fits is shown in Table 2 (RL/FM) and Table 3 (EL/FM) of the supplement. The relative and absolute model fit improved by allowing most parameters to vary between groups, suggesting distinct mechanisms of growth and change. Taken together across a set of model fit indices, the best fitting model solution for the growth trajectories of Receptive Language and Fine Motor is the model that allows all parameters, including structured residuals, to vary between groups. However, we note that the differences in model fit among the most complex candidates are marginal and not uniform across fit statistics. For Expressive Language and Fine Motor, the freed structured residuals did not add to a better fitting model, which is why, on balance of the fit indices, we consider the last model (with free error variances) to be the best fitting model. These models suggest that the groups differ in both person-specific and time-specific components of change: they differed in their mean intercept at FM, RL, and EL; their growth trajectories of all three skills (see Figure 3); and their time-specific residuals of the observed repeated measures. We note that in this multigroup modeling framework, the “best” models still showed relatively poor model fit overall. Exploratory inspection of the standardized residuals as well as modification indices did not suggest any clear candidates for model modification. Taken together with previous demonstrations that model fit in LGMs can become poor in moderate to small samples (DeRoche, 2009), we mention the mediocre model fit of the multigroup models as a point of caution but note that the adequate fit at the population level suggests the models can nonetheless be considered useful approximations of the developmental process.

**Figure 4.**
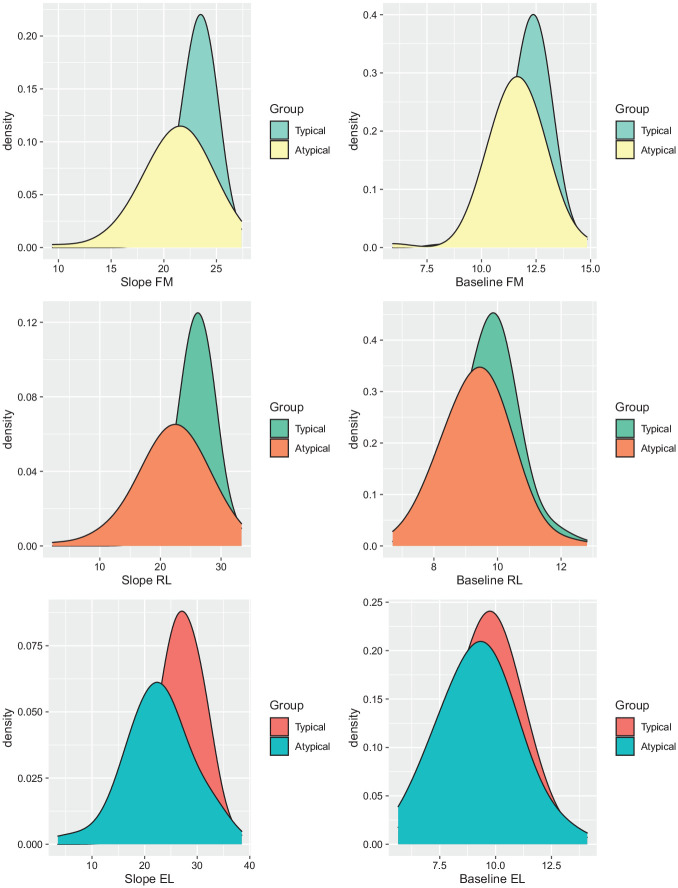
Group differences in model parameters for Fine Motor (FM), Receptive Language (RL), and Expressive Language (EL) for those children who develop typically versus those who develop atypically over time.

In conclusion, our findings suggest that the groups differ in both person-specific and time-specific components of change: we found group differences in the intercept and slope variance in both domains, with the atypically developing group displaying a much wider range of starting values and growth rates for EL, RL, and FM (see Figure 4). Also, the atypically developing group presented with generally lower scores and a slower growth rate for the assessed domains compared to the typical group. The groups did not differ in the covariance of intercepts between the domains, or in the cross-domain regression, for example, the intercept of FM regressed on the slope parameter of either language domain, or vice versa. The covariance between the slopes was similarly strong (~0.9), such that more rapid changes in language were usually associated with more rapid changes in motor abilities. We did not find evidence for differences in cross-domain coupling that drive group differences. In other words, the starting point in motor skills at 6 months was not associated with the rate of change in (either) language skills, or vice versa. The reported findings, combined with very strong correlations between the slopes, suggest that dynamic processes may amplify small differences between individuals at 6 months, resulting into large individual differences in developmental delays and symptomatology at 36 months. The width of the plotted curves in Figure 4 indicates the range of values per group (typical vs atypical) for FM, RL, and EL growth rates as well as FM, RL, and EL starting points. Figure 4 shows large differences in baseline scores with strong, positively correlated improvement over time in both domains. In other words, children who developed more rapidly in one domain also tended to develop more rapidly in the other. Highly correlated growth rates, showing more variance in the atypical group, suggest mechanisms that underlie the long-lasting phenotypic consequences of small early differences as these are amplified through these tightly linked trajectories of skills. This highlights the importance of studying such underlying dynamic processes (highly correlated growth rates) to work toward a mechanistic understanding of resulting phenotypic differences.

## Discussion

We examined parallel longitudinal changes in receptive and expressive language and motor skills in children diagnosed with autism and/or other developmental delays. The results indicated that development of both language and motor skills could be captured by a non-linear LGM, in which there was positive growth, but with considerable individual differences in both starting point as well as the rate of development over time in both domains. Changes in both language domains co-varied almost perfectly with changes in motor skills. There are multiple explanations for the high change–change correlation we found in this sample, including measurement artifacts such as assessment timing or shared underlying factors such as parenting style. While we are unable to disentangle what causes this, we regard it relevant to report this finding to complete the bigger picture of longitudinal dynamics that may amplify (small) early differences. Contrary to our hypothesis, we did not find compelling evidence for mutualistic coupling between these skills: Differences in children’s baseline motor skills did not seem to affect the rate of change in language skills, or vice versa. Infants who later receive a diagnosis of atypical development at age 3 years were not specifically characterized by increased or decreased coupling between language and motor skills compared to their peers. We observed group differences, however, in the variance of both baseline levels and trajectories of language and motor skills. The group of infants developing atypically showed a much wider range of trajectories and starting values than the group of typically developing children.

Our results suggest that the coupling between fine motor skills and language skills does not differ between infants who later receive a diagnosis and those who do not. Estes et al. (2015) showed a pattern of atypicalities in the sensorimotor domain at 6 months, which then shifted to the social-communication domain after 12 months of age, suggesting another temporal ordering of the interplay of these domains. Previous studies that also employed longitudinal approaches have suggested that slower growth in early fine motor skills was a significant predictor of later expressive language outcomes (Choi et al., 2018). Our results suggest that this predictive relationship cannot be mechanistically explained by group differences in how motor skills are actually linked to language development, for example, that they decouple during development or show different coupling strength. In line with Leonard et al.’s (2015) finding that the level of early motor skills did not predict the rate of growth in receptive language, we did not find a significant effect of such early motor levels between both receptive and expressive language. The divergence between our results and Leonard et al.’s results may be attributable to the differential contribution of fine versus gross motor skills to language development. Both motor domains are important for social interactions (Iverson, 2010), but it could very well be that gross motor skills, such as sitting and walking, contribute more to early language development than fine motor skills such as object manipulation and exploring (Koterba et al., 2014; LeBarton & Iverson, 2013). Adding to the Leonard et al. (2015) and Choi et al. (2018) study, we provide a more fine-grained picture of the longitudinal pathways that could eventually lead to phenotypic differences between typical and atypically developing. Future work should investigate dynamic coupling in different sets of developmental domains associated with autism, and possibly of higher temporal resolution, to evaluate these dynamics in different domains and on different timescales.

### Limitations and future directions

Several potential explanations for the absence of mutualistic coupling between language and motor skills in this cohort might be related to the constraints of the data, such as the small number of children developing atypically. This resulted in group comparisons of n = 165 children without a diagnosis versus only n = 74 children who go on to receive a diagnosis. Model misfit of this multigroup model suggests that it would be useful to replicate the study with a larger atypically developing sample. Of course, it is also a distinct possibility that differences in coupling do not help explain differences between atypically and typically developing children in the context of autistic phenotypes.

In addition, although we divide based on diagnostic status, it may be that a finer-grained division into subtypes of atypicality could be useful and may explain part or all of the differences in within-group variability. Landa et al. (2012), for example, found four distinct developmental trajectories across multiple developmental domains, which could very well mean that it is essential to distinguish between these subgroups to investigate differences in mutualistic coupling. Also, it is conceivable that mutualistic coupling between these domains occurs earlier or later in development, resulting in other mechanisms, such as self-feedback, once (or before) a certain equilibrium of skills is reached. It has been suggested, for example, that healthy development up until 6 months of age does not protect against atypical development, especially in infants with autism (Landa & Garrett-Mayer, 2006; Ozonoff et al., 2010). Future studies should further investigate the effect of age on cross-domain coupling in autism. Although exploring cross-domain coupling parameters across the life span is a fruitful framework for advancing our understanding of (a)typical development, an important limitation of this study is the use of only a single rater-reported measure to assess language and motor abilities in this cohort of infants on specific occasions. It should be noted that this is unlikely to capture the variability in language acquisition trajectories and induce a shared measurement error that could be addressed by using different multi-source (parent/clinician-rated) and observational measures.

Methodologically, we here implemented multigroup LGMs, a broad and flexible analytic strategy (e.g. Duncan & Duncan, 2004) with many strengths in capturing developmental patterns. It has been shown that the use of auxiliary variables outperforms other additions when imputing missing data. Limited by the variables we initially requested for secondary data analysis from the BASIS network, we were unable to apply this for the current study and therefore assume limited generalizability of our results. However, this is one among multiple potential analytic strategies, each of which may be able to shed complementary light on the challenges of understanding the complexities of typical and atypical development. Some particularly promising avenues include network analysis (e.g. Borsboom & Cramer, 2013), continuous time modeling (e.g. Driver & Voelkle, 2018), Gaussian mixture modeling (e.g. Fraley & Raftery, 1998), and various types of machine learning (Dwyer et al., 2018). As such, a whole field of “complexity science” is emerging, which marries novel conceptual frameworks with quantitative approaches able to capture non-linearities, discontinuities, interaction, and more (for an accessible introduction, see https://complexityexplained.github.io/). These advances might pave the way toward more formal (hierarchical) models connecting different levels of factors and mechanisms that drive atypical development and its consequences.

## Conclusion

In conclusion, our findings support the co-development of language skills and motor skill where less improvement in one domain is associated with less improvement in the other. However, we did not find that baseline ability in one domain is associated with change in the other, that is, no evidence for coupling. The infants in the current sample who eventually receive a diagnosis of atypical development do not differ from children who develop typically in terms of coupling between language and motor skills. We found that the later diagnosed group consistently displayed greater individual differences in both baseline scores and rates of change, suggesting the possibility of further latent heterogeneity. Such advances in understanding cross-domain interactions can eventually feed into the construction of novel explanatory models that are concerned with what drives specific developmental trajectories.

## Supplemental Material

sj-docx-1-aut-10.1177_13623613221086448 – Supplemental material for Longitudinal development of language and fine motor skills is correlated, but not coupled, in a childhood atypical cohort Click here for additional data file. Supplemental material, sj-docx-1-aut-10.1177_13623613221086448 for Longitudinal development of language and fine motor skills is correlated, but not coupled, in a childhood atypical cohort by Marie K Deserno, Delia Fuhrmann, Sander Begeer, Denny Borsboom, Hilde M Geurts and Rogier A Kievit in Autism

sj-docx-2-aut-10.1177_13623613221086448 – Supplemental material for Longitudinal development of language and fine motor skills is correlated, but not coupled, in a childhood atypical cohort Click here for additional data file. Supplemental material, sj-docx-2-aut-10.1177_13623613221086448 for Longitudinal development of language and fine motor skills is correlated, but not coupled, in a childhood atypical cohort by Marie K Deserno, Delia Fuhrmann, Sander Begeer, Denny Borsboom, Hilde M Geurts and Rogier A Kievit in Autism

## Notes

1. Note that the regression between baseline motor skills and language growth parameter is similar to the change parameter in Leonard et al. (2015). A simple, but not identical, re-estimation of the covariance in line with Leonard et al. between motor baseline score and language growth was also not significant (p = 0.675 for Fine Motor and Receptive Language; p = 0.296 for Fine Motor and Expressive Language), suggesting the difference is not solely due to model specification. However, as the present sample from the British Autism Study of Infant Siblings (BASIS) cohort is more than twice as large as the sample their results are based on, the results are not directly comparable to the Leonard et al. study. Although our findings here diverge somewhat from the Leonard et al. (2015) study, we note that they used gross motor skill at baseline, used a different instrument for language skills (the Vineland Adaptive Behavioral Scales; Sparrow & Cicchetti, 1989), and that in the current study the sample size was more than twice the size.
