# Supplementary material for: Longitudinal development of language and fine motor skills is correlated, but not coupled, in a childhood atypical cohort
Source: Autism. 2022 Apr 26;27(1):133–44. doi: 10.1177/13623613221086448 (PMC9806469; doi:10.1177/13623613221086448)
Supplement: sj-docx-1-aut-10.1177_13623613221086448 – Supplemental material for Longitudinal development of language and fine motor skills is correlated, but not coupled, in a childhood atypical cohort [file sj-docx-1-aut-10.1177_13623613221086448.docx]

***Supplement Table 1.*** Demographics and questionnaire scores for children classified as typically developing (TD) or atypically developing (AD) at 36 months.

|  | **Total sample** | **Classified as TD at 36m** | **Classified as AD at 36m** |
| --- | --- | --- | --- |
| N | 239 | 174 | 73 |
| Male [%] | 118 [49] | 73 [42] | 45 [62] |
| Female | 121 [51] | 101 [58] | 28 [38] |
| AOSI at 8m  [mean±SE] | 8.30±4.81 | 7.51±4.36 | 10.18±5.24 |
| ADOS at 36m | 5.49±4.67 | [3.86±3.31] | [9.2±5.18] |
| **Mullen Scales** |  |  |  |
| EL 6-9m | 9.51±2.30 | 9.58±2.26 | 9.38±2.45 |
| EL 14m | 14.44±2.89 | 14.86±2.77 | 13.41±2.82 |
| EL 24m | 24.26±5.17 | 24.91±4.66 | 22.51±5.88 |
| EL 36m | 35.39±6.57 | 37.49±4.65 | 30.57±7.28 |
| RL 6-9m | 9.70±2.18 | 9.90±2.05 | 9.22±2.33 |
| RL 14m | 14.90±3.12 | 15.28±3.08 | 14.00±2.77 |
| RL 24m | 25.92±4.73 | 26.97±3.57 | 23.09±5.71 |
| RL 36m | 34.48±6.12 | 36.25±4.19 | 30.43±7.76 |
| FM 6-9m | 11.98±2.67 | 12.31±2.62 | 11.11±2.57 |
| FM 14m | 18.65±2.18 | 19.04±1.79 | 17.88±2.73 |
| FM 24m | 25.26±2.95 | 25.18±2.26 | 24.18±3.92 |
| FM 36m | 34.67±5.12 | 36.07±4.25 | 31.44±5.48 |
| GM 6-9m | 11.72±2.15 | 11.88±2.02 | 11.36±2.40 |
| GM 14m | 18.90±3.64 | 19.29±3.66 | 18.16±3.60 |
| GM 24m | 27.24±3.64 | 27.66±S3.38 | 26.20±4.11 |
