# Supplementary material for: Longitudinal development of language and fine motor skills is correlated, but not coupled, in a childhood atypical cohort
Source: Autism. 2022 Apr 26;27(1):133–44. doi: 10.1177/13623613221086448 (PMC9806469; doi:10.1177/13623613221086448)
Supplement: sj-docx-2-aut-10.1177_13623613221086448 – Supplemental material for Longitudinal development of language and fine motor skills is correlated, but not coupled, in a childhood atypical cohort [file sj-docx-2-aut-10.1177_13623613221086448.docx]

***Supplementary Table 2****.* Model fit indices for the multigroup models with Receptive Language (RL) and Fine Motor (FM) with a stepwise addition of freed model parameters.

| Parameters | df | X^2^ | ΔX^2◊^ | p | AIC | BIC | RMSEA | CFI |
| --- | --- | --- | --- | --- | --- | --- | --- | --- |
| All constrained | 60 | 217.555 | 219.555 | <0.001 | 9213.379 | 9310.602 | 0.149 | 0.693 |
| Free intercepts and slopes | 56 | 160.317 | 164.097 | <0.001 | 9164.140 | 9275.253 | 0.127 | 0.792 |
| + free FM & RL intercept var | 54 | 139.902 | 163.249 | <0.001 | 9147.726 | 9265.783 | 0.130 | 0.790 |
| + free FM & RL slope var | 52 | 134.906 | 145.697 | <0.001 | 9146.729 | 9271.731 | 0.123 | 0.820 |
| + free error vars | 46 | 115.321 | 125.380 | <0.001 | 9139.145 | 9284.980 | 0.120 | 0.847 |
| + free structured residuals | 44 | 110.599 | 114.423 | <0.001 | 9138.422 | 9291.202 | 0.116 | 0.865 |

*Note.* df = degrees of freedom; AIC= Akaike Information Criterion; BIC= Bayesian Information Criterion; RMSEA = Root Mean Square Error of Approximation; CFI = Confirmatory Fit Index.

^◊^Maximum Likelihood Estimation-adjusted (with robust standard errors) Chi-Square difference test.

***Supplementary Table 3.*** Model fit indices for the multigroup models with Expressive Language (EL) and Fine Motor (FM) with a stepwise addition of freed model parameters.

| Parameters | df | X^2^ | ΔX^2◊^ | p | AIC | BIC | RMSEA | CFI |
| --- | --- | --- | --- | --- | --- | --- | --- | --- |
| All constrained | 60 | 233.102 | 242.222 | <0.001 | 9339.294 | 9436.518 | 0.160 | 0.681 |
| Free intercepts and slopes | 56 | 169.888 | 180.379 | <0.001 | 9284.080 | 9395.192 | 0.137 | 0.782 |
| + free FM & EL intercept var | 54 | 149.029 | 163.542 | <0.001 | 9267.222 | 9385.279 | 0.131 | 0.808 |
| + free FM & EL slope var | 52 | 145.142 | 159.410 | <0.001 | 9267.334 | 9392.336 | 0.132 | 0.812 |
| + free error vars | 44 | 129.106 | 143.068 | <0.001 | 9267.298 | 9420.078 | 0.138 | 0.827 |
|  |  |  |  |  |  |  |  |  |

*Note.* df = degrees of freedom; AIC= Akaike Information Criterion; BIC= Bayesian Information Criterion; RMSEA = Root Mean Square Error of Approximation; CFI = Confirmatory Fit Index.

^◊^Maximum Likelihood Estimation-adjusted (with robust standard errors) Chi-Square difference test.
